# Investigation of the Protective Effects of Phlorizin on Diabetic Cardiomyopathy in *db/db* Mice by Quantitative Proteomics

**DOI:** 10.1155/2013/263845

**Published:** 2013-02-25

**Authors:** Qian Cai, Baoying Li, Fei Yu, Weida Lu, Zhen Zhang, Mei Yin, Haiqing Gao

**Affiliations:** ^1^Key laboratory of Cardiovascular Proteomics, Qi-Lu Hospital of Shandong University, Jinan, Shandong 250012, China; ^2^Department of Geriatrics, Qi-Lu Hospital of Shandong University, 107 Wenhuaxi Road, Jinan, Shandong 250012, China

## Abstract

Patients with diabetes often develop hypertension and atherosclerosis leading to cardiovascular disease. However, some diabetic patients develop heart failure without hypertension and coronary artery disease, a process termed diabetic cardiomyopathy. Phlorizin has been reported to be effective as an antioxidant in treating diabetes mellitus, but little is known about its cardioprotective effects on diabetic cardiomyopathy. In this study, we investigated the role of phlorizin in preventing diabetic cardiomyopathy in *db/db* mice. We found that phlorizin significantly decreased body weight gain and the levels of serum fasting blood glucose (FBG), triglycerides (TG), total cholesterol (TC), and advanced glycation end products (AGEs). Morphologic observations showed that normal myocardial structure was better preserved after phlorizin treatment. Using isobaric tag for relative and absolute quantitation (iTRAQ) proteomics, we identified differentially expressed proteins involved in cardiac lipid metabolism, mitochondrial function, and cardiomyopathy, suggesting that phlorizin may prevent the development of diabetic cardiomyopathy by regulating the expression of key proteins in these processes. We used ingenuity pathway analysis (IPA) to generate an interaction network to map the pathways containing these proteins. Our findings provide important information about the mechanism of diabetic cardiomyopathy and also suggest that phlorizin may be a novel therapeutic approach for the treatment of diabetic cardiomyopathy.

## 1. Introduction

The prevalence of diabetes mellitus is rapidly increasing worldwide [1]. Patients with diabetes often develop hypertension and atherosclerosis leading to cardiovascular complications. However, some diabetic patients develop heart failure without hypertension and coronary artery disease [2]. This phenomenon was first described by Rubler et al. and was termed “diabetic cardiomyopathy” [3]. Diabetic cardiomyopathy is characterized by structural and functional changes in the heart, such as elevated left ventricular (LV) mass, myocardial fibrosis, and abnormal diastolic function [4, 5]. However, the mechanistic details of diabetic cardiomyopathy remain unclear, and this disease has not yet been sufficiently studied.

 Phlorizin (phloretin-2′-O-glucoside), a dihydrochalcone derived from apple peels, is a known antioxidant [6]. The main pharmacological property of phlorizin is to produce renal glycosuria and block intestinal glucose absorption through inhibition of sodium/glucose cotransporters in the kidney and intestine [7]. Although cardioprotective benefits of phlorizin have been reported, little is known about the effect of phlorizin on cardiac damage in type 2 diabetes mellitus (T2DM).

In this study, we used phlorizin to treat T2DM in *db/db* mice. These mice exhibit symptoms such as hyperglycemia, obesity, insulin resistance, and renal damage, which occurs after 10–20 weeks of sustained hyperglycemia [8, 9]. Additionally, we used a quantitative proteomic assay, isobaric tag for relative and absolute quantitation (iTRAQ), combined with liquid chromatography-tandem mass spectrometry (LC-MS/MS) to identify and characterize the protein profiles of phlorizin-treated and untreated *db/db* mice. The iTRAQ technique has been widely used to tag peptides for multiplexed protein quantification and provides increased experimental throughput and lower variability [10, 11].

## 2. Materials and Methods

### 2.1. Experimental Animal Treatment

Male C57BLKS/J *db/db* and *db/m* mice (*n* = 24, 7 weeks old) were purchased from the Model Animal Research Center at Nanjing University (Jiangsu, China). All mice were housed in wire-bottomed cages and received laboratory pellet chow and tap water *ad libitum* in a constant environment (room temperature 22 ± 1.6°C, room humidity 55 ± 5%) with a 12 h-light, 12 h-dark cycle. All experimental protocols were verified and approved by the Animal Ethics Committee of Shandong University. C57BLKS/J *db/m* mice were used as a control group, which were administered normal saline solution (*n* = 8). The *db/db* mice were randomly divided into two groups: the vehicle-treated diabetic group (DM, *n* = 8), which were administered normal saline solution, and the phlorizin-treated diabetic group (DMT, *n* = 8), which were treated with 20 mg/kg phlorizin. Phlorizin (purity >98%, Jianfeng Inc., Tianjin, China) was dissolved in normal saline solution and administered intragastrically from week 8 to week 18 without hypoglycemic therapy. Animals were weighed each week. At the end of the study, all mice were fasted overnight. Fasting blood was collected before sacrifice to measure fasting blood glucose (FBG), blood triglycerides (TG), and blood total cholesterol (TC) using an Automatic Biochemistry and Analysis Instrument (DVI-1650, Bayer, Germany). Specific fluorescence determinations of serum advanced glycation end products (AGEs) were performed using a fluorescence spectrophotometer (Hitachi F-2500, Japan) by measuring 440 nm emissions after excitation at 370 nm. The hearts of the mice were immediately dissected. Tissue and sera were kept at −80°C until further analysis.

### 2.2. Histological Examination and Ultrastructure Observation

The LV myocardium was fixed in 4% paraformaldehyde and embedded in paraffin. Five-millimeter-thick sections were cut, stained with hematoxylin-eosin (H&E), and examined by light microscopy. Additionally, part of the LV free wall was fixed in 3% glutaraldehyde. Ultrathin sections cut from embedded blocks were stained with uranyl acetate and lead citrate and examined with an H-800 electron microscope (Hitachi, Japan).

### 2.3. iTRAQ Proteomic Analysis

Heart tissue (50 mg) from each of four mice per group was prepared and digested with trypsin, as previously described [12]. A total of 60 *μ*g of peptides from each group were labeled with iTRAQ reagents following the manufacturer's instructions (Applied Biosystems). The control group peptides were labeled with Reagent 114; the DMT group, Reagent 116; and the DM group, Reagent 117. The labeled samples were then separated into 10 fractions using PolySULFOETHYL A strong cation-exchange (SCX) columns (4.6 × 100 mm 5 *μ*, 200 Å, PolyLC). Mass spectrometric analysis was performed using a micro-liquid chromatography system (MDLC, GE Healthcare, Pittsburgh, PA, USA) and an LTQ Velos ion trap mass spectrometer (ThermoFinnigan, San Jose, CA, USA). 

### 2.4. Protein Pathway Analysis

 Differentially expressed genes were analyzed using Ingenuity Pathway Analysis (IPA, Ingenuity Systems, http://www.ingenuity.com/). The data packet containing the differentially expressed proteins identified in the iTRAQ experiment was converted by IPA to “fold change” and uploaded into IPA. Each identifier was mapped to its corresponding gene object in the Ingenuity Pathways Knowledge Base. 

### 2.5. Western Blotting Analysis

Proteins were separated by SDS-polyacrylamide gel electrophoresis and transferred onto polyvinylidene difluoride membranes as previously described [13]. Membranes were subsequently probed with antibodies against calnexin (1 : 500 dilution, Abcam), integrin-linked protein kinase (1 : 1000 dilution, Santa Cruz Biotechnology), or GAPDH (1 : 5000 dilution, Santa Cruz Biotechnology) overnight at 4°C, which was then followed by incubation with secondary antibody for 2 h. The band intensity was quantified using VisionWorks LS image acquisition and analysis software (UVP, Upland, CA, USA).

### 2.6. Statistical Analysis

The data are presented as the mean ± standard deviation. Statistical comparisons among the three experimental groups were made using the unpaired Student's *t*-test and one-way ANOVA. A value of *P* < 0.05 was considered statistically significant.

## 3. Results

### 3.1. General Metabolic Parameters and AGEs

**Figure 1:**
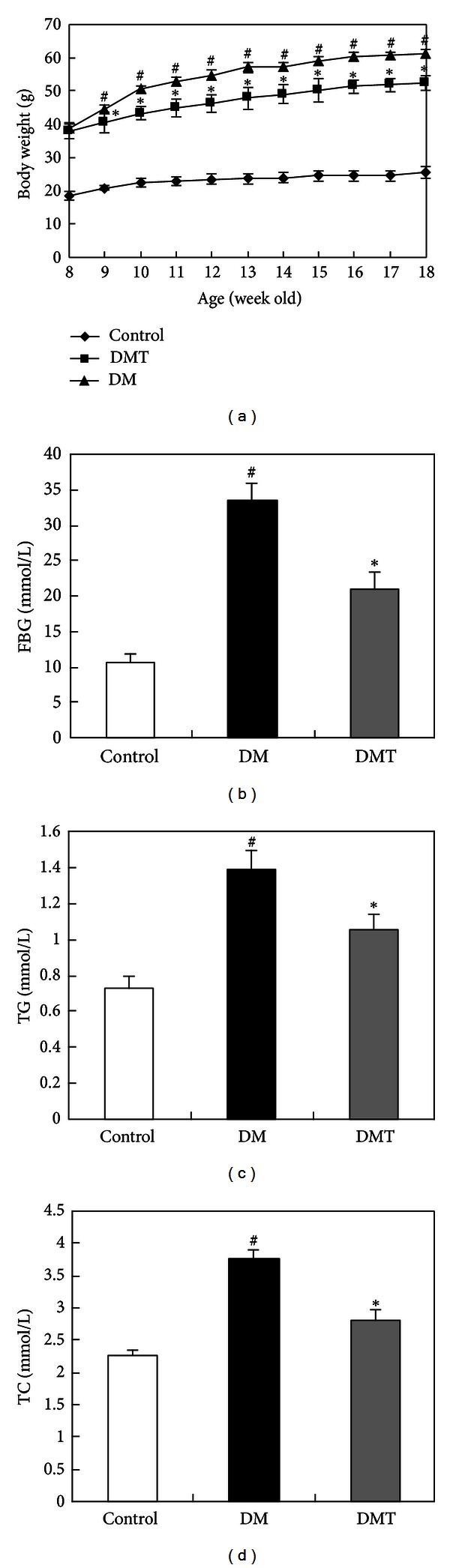
General metabolic parameters. (a) Body weight change of mice in the control, DM, and DMT groups. ((b), (c), (d)) Serum FBG, TG, and TC were measured after 10 weeks.  ^#^ *P* < 0.05 and indicates a significant difference between the control and DM group;  *P < 0.05 and indicates a significant difference between the DMT and DM group (*n* = 8). DM: vehicle-treated diabetic group; DMT: diabetic group treated with 20 mg/kg phlorizin; FBG: fasting blood glucose; TG: triglycerides; TC: total cholesterol.

**Figure 2:**
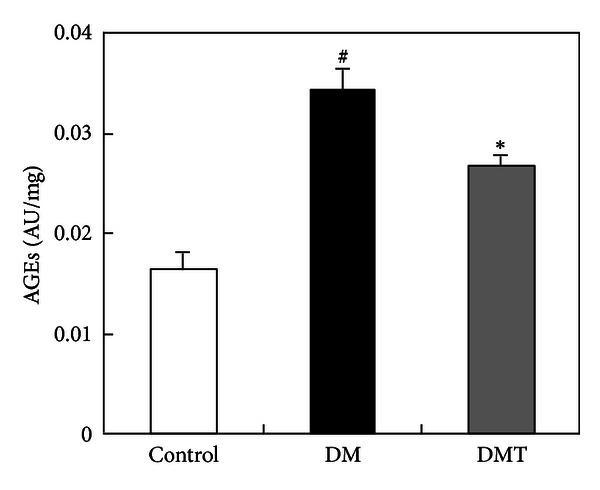
The effect of phlorizin on AGE levels in blood.  ^#^ *P* < 0.05, and indicates a significant difference between the control and DM group;  *P < 0.05, and indicates a significant difference between the DMT and DM group (*n* = 8). DM: vehicle-treated diabetic group; DMT: diabetic group treated with 20 mg/kg phlorizin; AGEs: advanced glycation end products.

During the observation period, the DM and DMT groups gained substantially more weight than the control group. Nevertheless, phlorizin treatment significantly reduced body weight gain in *db/db* mice at the second week after phlorizin administration (Figure 1(a)). After 10 weeks, serum FBG, TG, and TC levels in the DM group were significantly higher than those in the control group. However, phlorizin treatment dramatically reduced these values in the DMT group compared with the DM group (Figures 1(b), 1(c), and 1(d)). In addition, *db/db* mice had significantly elevated serum AGE levels. After phlorizin treatment, AGE levels in *db/db* mice were reduced (Figure 2).

### 3.2. Histological and Ultrastructural Observation

**Figure 3:**
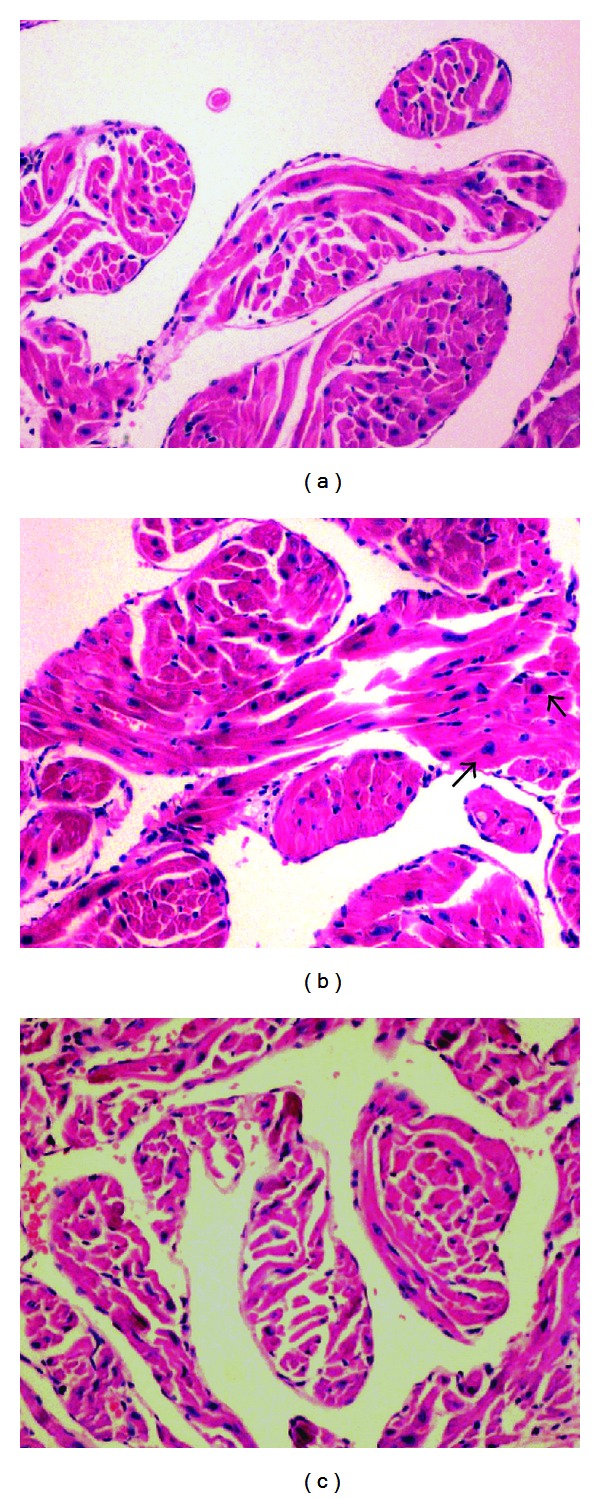
H&E staining of LV myocardium after 10 weeks: (a) control group, (b) DM group, and (c) DMT group. Magnification, 400x. Arrows indicate the hypertrophic cardiomyocytes with enlarged nuclei.

On H&E-stained sections, the DM group exhibited significant myocardial hypertrophy and myofiber disarray accompanied by damaged nuclei and increased degeneration. However, phlorizin treatment attenuated this cardiomyocyte hypertrophy to a level similar to the control group (Figures 3(a), 3(b), and 3(c)).

**Figure 4:**
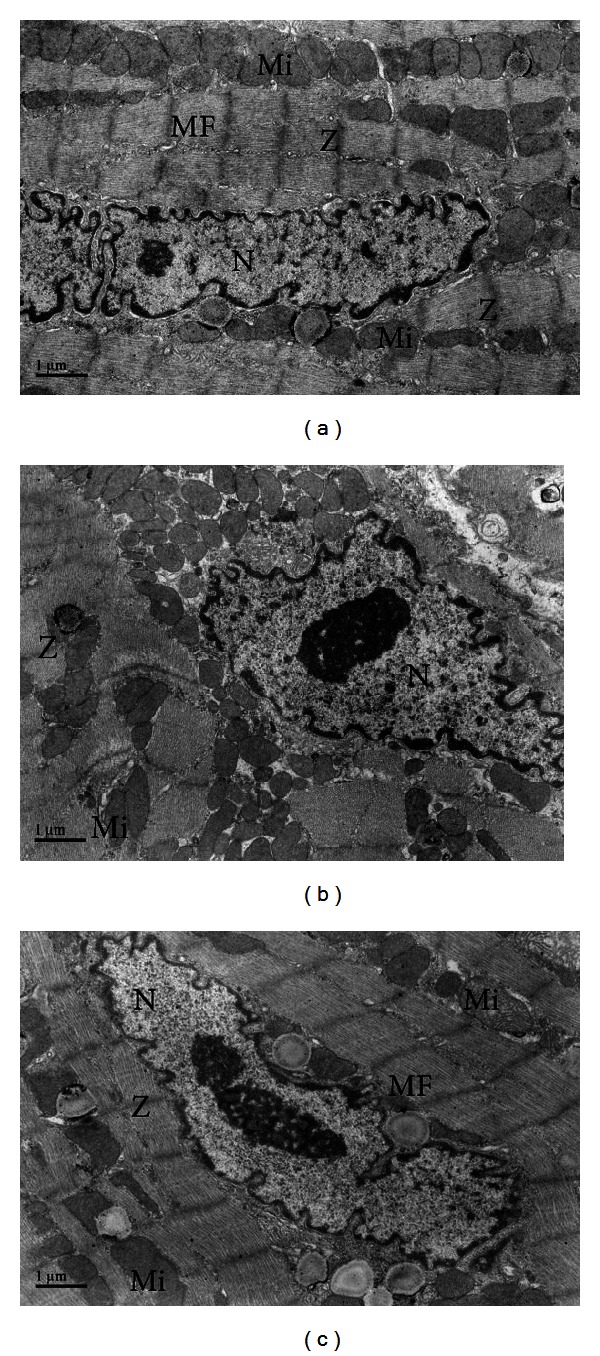
Electron microscopic examination of nuclei, mitochondria, sarcomeres, and myofibrils in the LV myocardium after 10 weeks: (a) control group, (b) DM group, and (c) DMT group. N: nuclei; Mi: mitochondria; Z: sarcomere; MF: myofibrils.

Myocardial ultrastructure could be visualized by electron microscopy (Figures 4(a), 4(b), and 4(c)). In the control group, the myofibrils were arranged in a striated pattern, and the mitochondria were positioned in rows along the myofibrils. Although the sarcomere was of the same length, some cardiomyocyte mitochondria in the DM group showed cristae loss. Large areas of the myocardium exhibited a complete disruption of myofibril and mitochondrial arrangements. The shape of the nuclei was altered, and the nuclear membrane was disrupted. However, due to the protective effect of phlorizin in the DMT group, the number of degenerated mitochondria was significantly decreased, and the myofibril disorder was markedly attenuated.

### 3.3. iTRAQ Proteomics Profiling

Using the iTRAQ approach, we analyzed the effect of phlorizin on the myocardial protein profile of *db/db* mice. A total of 1627 proteins were identified. Of the 113 differentially expressed proteins, 29 were elevated in the DM group compared with the control group but were still decreased by phlorizin treatment. An additional 84 proteins were decreased in the DM group compared with the control group, but these were restored by the phlorizin treatment (see Supplementary Material available online at http://dx.doi.org/10.1155/2013/263845).

**Figure 5:**
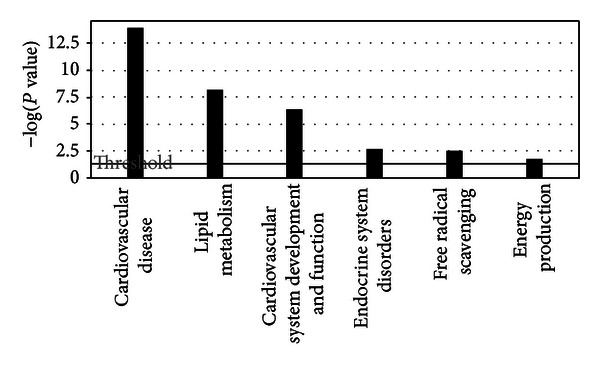
Top biofunctional processes associated with the differentially expressed proteins generated by IPA.

**Figure 6:**
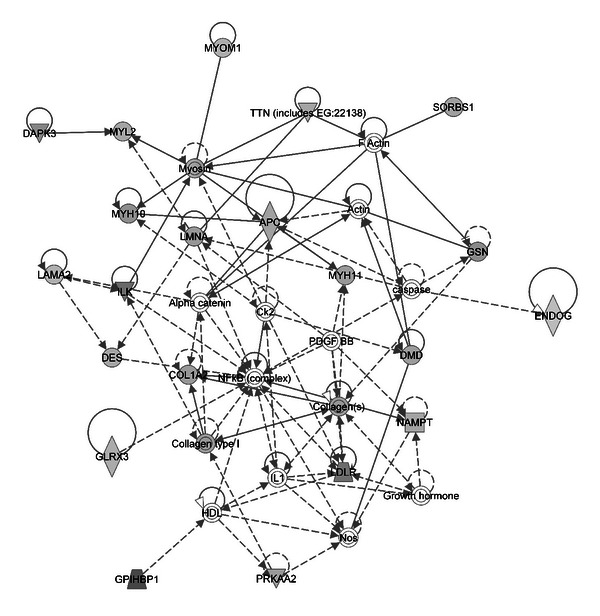
The top network in which the differentially expressed proteins might be involved. The network is for cardiovascular disease, lipid metabolism, and organ development processes.

We used IPA software to conduct gene ontology analysis and to classify the molecular functions of significantly altered proteins.  Figure 5 shows the top-ranked biological functions, including lipid metabolism and energy production, which are biological processes altered during diabetic cardiomyopathy. The top protein network was generated by pathway analysis of differentially expressed proteins (Figure 6). There was a cluster of 35 proteins in the network, of which 24 are included on our list. These proteins are likely to be involved in biological processes such as lipid metabolism, mitochondrial function, and cardiomyopathy. 

### 3.4. Functional Classification of Proteins Involved in Metabolic Disorders in *db/db* Mice Detected by iTRAQ 

#### 3.4.1. Altered Proteins in Cardiac Lipid Metabolism

Lipotoxicity occurring with T2DM and obesity impairs cardiac lipid metabolism [14]. The identified proteins associated with cardiac lipid metabolism are listed in Table 1. Proteins upregulated after phlorizin treatment included microsomal triglyceride transfer protein (Mttp), nicotinamide phosphoribosyltransferase (Nampt), tyrosine-protein phosphatase nonreceptor type 11 (Ptpn11), low-density lipoprotein receptor (LDLr), protein-tyrosine phosphatase-like member B (Ptplb), and sorbin and SH3 domain-containing protein 1 (Sorbs1). However, glycosylphosphatidylinositol-anchored high-density lipoprotein-binding protein 1 (Gpihbp1), which was initially identified as an HDL-binding protein involved in reversing cholesterol transport, was downregulated in the DMT group compared with the DM group. 

#### 3.4.2. Altered Proteins Related to Myocardial Mitochondria

 Diabetic cardiomyopathy is usually associated with abnormal energy production due to impaired mitochondrial function. We detected several proteins related to myocardial mitochondria (Table 1). Proteins that were significantly upregulated in the DMT group compared with the DM group include the following: 5′-AMP-activated protein kinase catalytic subunit alpha-2 (Prkaa2), endonuclease G (EndoG), and NADH dehydrogenase (ubiquinone) iron-sulfur protein 6 (Ndufs6). Other proteins such as apoptosis-inducing factor 2 (Atpaf2), ATP synthase mitochondrial F1 complex assembly factor 2 (Aifm2), and lipoic acid synthase (Lias) were significantly downregulated in the DMT group compared with the DM group. 

#### 3.4.3. Altered Proteins Involved in Cardiomyopathy

Several factors contribute to diabetic cardiomyopathy, including myocardial hypertrophy, elevated wall-thickness-to-chamber ratios, and increased stiffness of the LV wall [15]. We found that several proteins associated with cardiac contraction and diastolic function were decreased in the DM group and reversed after phlorizin treatment (Table 1). For example, the cytoskeletal protein titin (Ttn) and death-associated protein kinase 3 (DAPK3 or ZIPK) were upregulated in the DMT group compared with the DM group. 

There were also some differentially expressed proteins on this list. Missense mutations or small deletions in some genes, for example, desmin (Des), integrin-linked protein kinase (Ilk), myosin regulatory light chain 2 (My12), dystrophin (Dmd), gelsolin (Gsn), lamin A/C (Lmna), and laminin subunit *α*-2 (Lama2), have been linked to cardiomyopathy. These proteins were upregulated in the DMT group compared with the DM group, indicating that diabetic cardiomyopathy improved after phlorizin treatment. We also listed the differentially expressed proteins involved in cardiac hypertrophy, including glutaredoxin-3 (Glrx3) and collagen alpha-2(I) chain (Col1a2). These proteins were upregulated in the DMT group compared with the DM group. Other important proteins involved in heart development and disease that were altered in *db/db* mice and reversed after phlorizin treatment include adenomatous polyposis coli (Apc), calnexin (Canx), myomesin-1 (Myom1), and voltage-dependent L-type calcium channel subunit beta-2 (Cacnb2). 

### 3.5. Validation of iTRAQ Data for Selected Candidate Proteins

**Figure 7:**
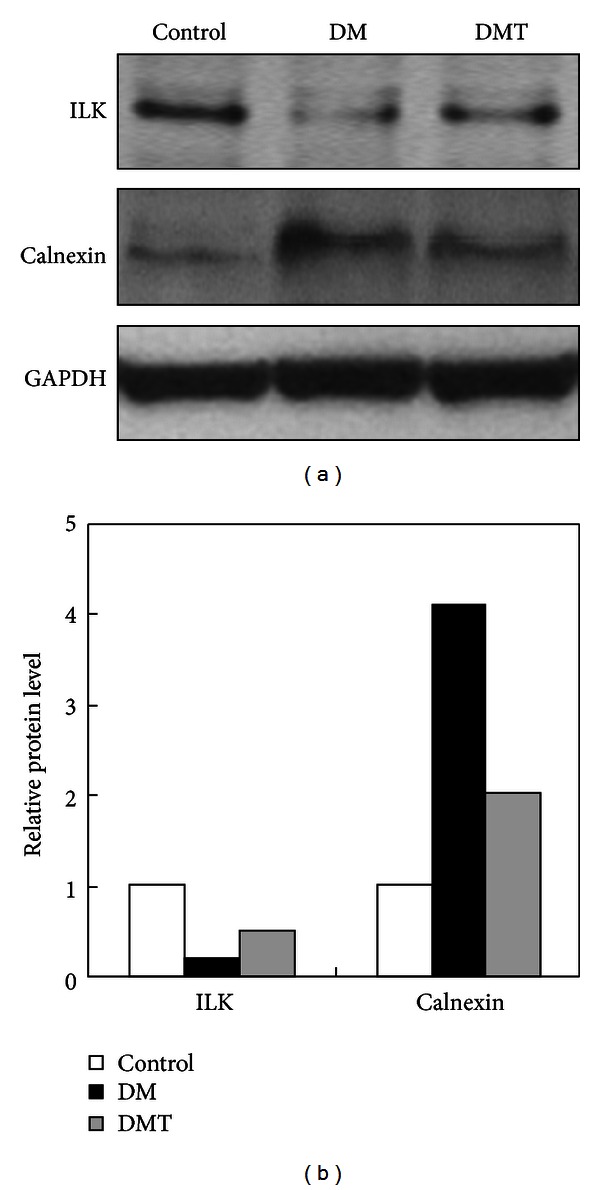
(a) Western blot validation of the differentially expressed proteins integrin-linked protein kinase (Ilk) and Calnexin. (b) Quantification of band intensity using VisionWorks LS image acquisition and analysis software.

We selected two proteins for Western blot analysis to validate the iTRAQ data. As shown in Figure 7(a), calnexin was found to be decreased, whereas integrin-linked protein kinase was increased in the DMT group compared with the DM group. Quantification of band intensity showed that the results from density of bands are almost consistent with the iTRAQ data (Figure 7(b)). This indicates that the iTRAQ data are reliable.

## 4. Discussion

Diabetic cardiomyopathy accompanying T2DM is a complicated disorder caused by multifactorial pathology including altered cardiac energy metabolism and increased oxidative stress [16]. Obesity is associated with high levels of circulating fatty acids, which result in increased fatty acid uptake and TG accumulation in the myocardium. Furthermore, increased oxygen damage and generation of reactive oxygen species (ROS) augment cardiac damage [17]. Thus, the normalization of cardiac energy metabolism and reduction in oxidative stress may be important factors in the treatment of diabetic cardiomyopathy.

Phlorizin has been reported to have an antidiabetic effect due to its antioxidant properties [18]. In this study, we observed that the levels of serum FBG, TG, and TC in the DM group were dramatically elevated compared with the control group and that the oral administration of phlorizin significantly reduced these levels. These results suggest that phlorizin may be able to prevent diabetes and its complications by lowering blood FBG, TG, and TC levels.

Based on the iTRAQ data and the IPA results, protein expression involved in cardiac lipid metabolism seems to be markedly stimulated by phlorizin. In diabetic patients, elevated circulating fatty acids and TG, together with hyperinsulinemia, augment cardiac uptake of fatty acids and storage of TG [19, 20]. However, when fatty acid delivery overtakes the metabolic capacity of cardiomyocytes, impaired cardiac lipid homeostasis ultimately leads to lipotoxic cardiomyopathy [21]. Among the proteins related to cardiac lipid metabolism that we identified here, Mttp and Namtp deserve additional attention. Studies have shown that Mttp expression is increased in obese mouse hearts compared with healthy ones. Upregulation of cardiac Mttp expression prevents myocardial lipid accumulation when the supply of fatty acids exceeds the need for energy generation [22]. Namtp, also known as visfatin, is elevated in patients with T2DM, obesity, and cardiovascular disease. The elevated level of circulating visfatin may be associated with insulin resistance and metabolic syndrome [23]. Mttp and Namtp were both upregulated in the DMT group compared with the DM group, suggesting that phlorizin may modulate cardiac lipid metabolism by lowering the level of circulating fatty acids and attenuating cardiac lipid accumulation.

In a diabetic heart, glucose utilization is diminished. Instead, the heart relies almost exclusively on fatty acids for ATP generation. Increased fatty acid uptake by the heart reduces energy efficiency by inducing mitochondrial damage [16]. In this study, we identified several important proteins connected to mitochondrial structure and function. The altered expression of these proteins may cause deleterious effects on cells, resulting in cardiac energy deficit and cardiomyopathy. Here, we found that the phlorizin treatment reversed the expression of these proteins, suggesting a correlation between the cardioprotective effect of phlorizin and proteins involved in mitochondrial energy production. 

Oxidative stress has been implicated in the pathogenesis of diabetes. A high rate of fatty acid oxidation also causes a pathological ROS accumulation, which leads to mitochondrial damage in cardiomyocytes [17]. Several factors contribute to ROS production in T2DM, such as AGEs [24]. In this study, we found that phlorizin treatment can significantly lower plasma AGE levels in *db/db* mice, which is consistent with its antioxidative ability to decrease ROS generation.

The development of diabetic cardiomyopathy has been divided into two phases: early metabolic alterations and later myocardial degenerative changes [25]. These irreversible pathological alterations include an increased stiffness of the LV wall, the accumulation of connective tissue and insoluble collagen, and abnormalities of various proteins [26]. Among the identified proteins that are involved in cardiac remodeling, titin was upregulated in the DMT group compared with the DM group. Recent observations have shown that the intrasarcomeric protein titin can alter myocardial diastolic stiffness through a number of different mechanisms such as isoform shifts, phosphorylation by protein kinase G or protein kinase A and titin-actin interactions at the Z-disc [27]. Another upregulated protein was Dapk3, also called ZIPK. Recent studies have identified smooth muscle myosin regulatory light chain and the regulatory subunit of the smooth muscle myosin light chain phosphatase as substrates for ZIPK [28]. This evidence suggests a key role for ZIPK in the regulation of cardiac contractility [29]. Here, our results demonstrate a cardioprotective role for phlorizin through the regulation of genes modulating cardiac contraction and diastolic function.

In conclusion, for the first time, the present study has established the quantitative iTRAQ profile of global cardiac proteins using a *db/db* diabetic mouse model treated with or without phlorizin. We found that phlorizin treatment may protect against diabetic cardiomyopathy by modulating cardiac lipid and energy metabolism and altering the expression of a set of proteins involved in cardiac damage. We also observed that phlorizin treatment significantly decreased body weight, blood glucose, blood TG, and blood TC. These findings suggest that, in the future, phlorizin may be utilized as a novel effective therapeutic approach for the treatment of diabetic cardiomyopathy.

## Supplementary Material

The description along with the Supplementary Material is as following: An accession no. is a unique identifier given to protein sequence when it is submitted to a sequence database. Theoretical molecular weight (Da) or PI are based on the amino acid sequence of the identified proteins.

## Figures and Tables

**Table 1:** Functional classification of altered proteins related to cardiac lipid metabolism, mitochondrial function, and cardiomyopathy.

Accession no.	Symbol	Protein name	Molecular weight (Da)	PI	iTRAQ ratio (DM/C)	iTRAQ ratio (DMT/DM)
Cardiac lipid metabolism						

IPI00785217	LDLr	Low-density lipoprotein receptor	94947.38	4.82	0.22	3.05
IPI00943405	Mttp	Microsomal triglyceride transfer protein large subunit	100750.94	7.51	0.4	1.83
IPI00655029	Sorbs1	Sorbin and SH3 domain-containing protein 1	103977.89	5.65	0.46	1.83
IPI00320188	Nampt	Nicotinamide phosphoribosyltransferase	55446.82	6.69	0.53	1.78
IPI00133956	Gpihbp1	Glycosylphosphatidylinositol-anchored high density lipoprotein-binding protein 1	24566.22	4.81	5.71	0.19
IPI00124411	Ptplb	Protein-tyrosine phosphatase-like member B	28402.43	9.59	0.23	3.95
IPI00316479	Ptpn11	Tyrosine-protein phosphatase nonreceptor type 11	68034.75	6.87	0.16	2.95

Mitochondrial components and function						

IPI00882331	Lias	Lipoyl synthase, mitochondrial	32173.8	8.48	2.63	0.49
IPI00128345	Ndufs6	NADH dehydrogenase [ubiquinone] iron-sulfur protein 6, mitochondrial	13019.78	8.86	0.59	1.55
IPI00114840	Endog	Endonuclease G, mitochondrial	32190.74	9.56	0.39	1.78
IPI00336348	Atpaf2	ATP synthase mitochondrial F1 complex assembly factor 2	34287.29	6.35	1.68	0.65
IPI00276157	Aifm2	Apoptosis-inducing factor 2	41355.63	8.16	2.58	0.45
IPI00929796	Prkaa2	5′-AMP-activated protein kinase catalytic subunit alpha-2	62022.37	7.94	0.43	1.81

Cardiomyopathy						

IPI00896736	Apc	Adenomatosis polyposis coli	310880.6	7.44	2.39	0.45
IPI00315550	Glrx3	Glutaredoxin-3	37778.42	5.42	0.35	1.61
IPI00130102	Des	Desmin	53497.99	5.21	0.3	1.7
IPI00116668	Ilk	Integrin-linked protein kinase	51373.15	8.3	0.18	3.15
IPI00756257	Ttn	Titin	3906489.35	5.91	0.34	1.69
IPI00555015	Myl2	Myosin regulatory light chain 2, ventricular/cardiac muscle isoform	18864.35	4.86	0.38	1.73
IPI00117846	Dapk3	Death-associated protein kinase 3	51421.9	8.91	0.33	2.45
IPI00828253	Dmd	Dystrophin	425831.77	5.66	0.46	1.88
IPI00759948	Gsn	Gelsolin	80762.65	5.52	0.41	2.33
IPI00222188	Col1a2	Collagen alpha-2(I) chain	129556.97	9.27	0.17	2.05
IPI00620256	Lmna	Isoform A of Lamin-A/C	74237.82	6.54	0.18	2.05
IPI00874362	Lama2	Laminin subunit alpha-2	342781.06	5.78	0.32	1.51
IPI00119618	Canx	Calnexin	67278.1	4.5	1.77	0.64
IPI00626655	Myom1	Myomesin-1	185464.52	5.83	0.48	1.54
IPI00420996	Cacnb2	Voltage-dependent L-type calcium channel subunit beta-2	64714.53	9.17	0.59	1.69
