# Supplementary material for: Investigation of the Protective Effects of Phlorizin on Diabetic Cardiomyopathy in db/db Mice by Quantitative Proteomics
Source: J Diabetes Res. 2013 Feb 25;2013:263845. doi: 10.1155/2013/263845 (PMC3647560; doi:10.1155/2013/263845)
Supplement: Supplementary file 1 — The description along with the Supplementary Material is as following: An accession no. is a unique identifier given to protein sequence when it is submitted to a sequence database. Theoretical molecular weight (Da) or PI are based on the amino acid sequence of the identified proteins. [file 263845.f1.docx]

**Table 1. Differentially expressed proteins as reversed by phlorizin identified by iTRAQ (Upregeulation)**

| Accession no. | Protein name | | Molecular weight (Da) | PI | Protein expression ratio (DM/C) | Protein expression ratio (DMT/DM) | Function |
| --- | --- | --- | --- | --- | --- | --- | --- |
| IPI00880521 | Solute carrier family 13 | | 14485.73 | 6.62 | 15.33 | 0.52 | copper ion transmembrane transporter activity |
| IPI00956780 | Putative uncharacterized protein Sorbs2 | | 78155.37 | 9.42 | 19.67 | 0.29 | zinc ion binding |
| IPI00131334 | Cation channel sperm- associated protein 1 | | 78705.89 | 6.8 | 5.53 | 0.61 | voltage-gated calcium channel activity |
| IPI00128152 | Multidrug resistance protein 1 | | 140993.72 | 8.51 | 7.45 | 0.31 | ATP binding |
| IPI00622235 | Transitional endoplasmic reticulum ATPase | | 89322.04 | 5.14 | 3.02 | 0.65 | ATPase activity |
| IPI00315974 | Bcl10-interacting CARD protein | | 20936.15 | 8.24 | 3.37 | 0.55 | regulation of apoptotic process |
| IPI00136653 | Syntaxin-8 | | 26924.83 | 4.92 | 3.21 | 0.52 | transport |
| IPI00845729 | Isoform 2 of Acyl-CoA-binding domain-containing protein 5 | | 52369.89 | 5.16 | 2.83 | 0.58 | fatty-acyl-CoA binding |
| IPI00153421 | Cytoplasmic dynein 1 light intermediate chain 1 | | 56614.29 | 6.01 | 2.81 | 0.52 | ATP binding |
| IPI00882331 | Putative uncharacterized protein, Lias | | 32173.8 | 8.48 | 2.63 | 0.49 | response to oxidative stress |
| IPI00622283 | Isoform 1 of Exportin-5 | | 136973.27 | 5.57 | 2.42 | 0.52 | ATP binding |
| IPI00831326 | Isoform 1 of FAD synthase | | 54766.67 | 6.19 | 2.32 | 0.53 | ATP binding |
| IPI00276157 | Isoform 2 of Apoptosis-inducing factor 2 | | 41355.63 | 8.16 | 2.58 | 0.45 | oxidoreductase, which may play a role in mediating a p53/TP53-dependent apoptosis response. |
| IPI00119618 | Calnexin | | 67278.1 | 4.5 | 1.77 | 0.64 | calcium ion binding |
| IPI00649931 | Isoform 2 of CMRF35-like molecule 9 | | 40233.01 | 6.12 | 1.83 | 0.61 | receptor activity |
| IPI00938437 | Aflatoxin B1 aldehyde reductase member 2 | | 40612.21 | 8.36 | 1.75 | 0.64 | alditol:NADP+ 1-oxidoreductase activity |
| IPI00336348 | ATP synthase mitochondrial F1 complex assembly factor 2 | | 34287.29 | 6.35 | 1.68 | 0.65 | may play a role in the assembly of the F1 component of the mitochondrial ATP synthase (ATPase) |
| IPI00134353 | Nucleolar protein 3 | | 24567.82 | 4.03 | 1.63 | 0.66 | regulation of apoptotic process |
| IPI00896736 | Adenomatosis polyposis coli | | 310880.6 | 7.44 | 2.39 | 0.45 | positive regulation of cell division, migration and differentiation. |
| IPI00133956 | Glycosylphosphatidylinositol-anchored high density lipoprotein-binding protein 1 | | 24566.22 | 4.81 | 5.71 | 0.19 | lipid transport, triglyceride homeostasis |
| IPI00316989 | Isoform 1 of Mammalian ependymin-related protein 1 | | 25485.16 | 7.51 | 1.79 | 0.58 | calcium ion binding |
| IPI00221998 | Rik Adipocyte plasma membrane-associated protein | | 46434.43 | 5.97 | 1.62 | 0.63 | arylesterase activity |
| IPI00621460 | AP-1 complex subunit gamma-1 | | 91350.47 | 6.36 | 1.54 | 0.66 | protein transporter activity |
| IPI00885610 | Secretory carrier-associated membrane protein 3 | | 38458.31 | 7.55 | 5.78 | 0.17 | intracellular protein transport |
| IPI00128069 | UPF0729 protein C18orf32 homolog | | 8035.57 | 9.44 | 1.57 | 0.64 | signal transducer activity |
| IPI00881383 | Putative uncharacterized protein, Rpl9 | 9279.85 | | 10.74 | 3.12 | 0.32 | rRNA binding |
| IPI00556889 | Isoform 2 of Polyribonucleotide nucleotidyltransferase 1, mitochondrial | | 58923.03 | 7.83 | 1.51 | 0.65 | miRNA binding |
| IPI00410796 | Protein ITFG3 | | 60575.99 | 5.38 | 1.56 | 0.63 | unclear |
| IPI00885274 | Transmembrane protein 141 | | 11676.44 | 7.94 | 2.55 | 0.36 | unclear |

**Table 2. Differentially expressed proteins as reversed by phlorizin identified by iTRAQ (Downregeulation)**

| Accession no. | Protein name | Molecular weight (Da) | PI | Protein expression ratio (DM/C) | Protein expression ratio (DMT/DM) | Function |
| --- | --- | --- | --- | --- | --- | --- |
| IPI00314254 | B ankyrin repeat and death domain-containing protein homolog | 52161.27 | 6.82 | 0.01 | 4.41 | signal transduction |
| IPI00875673 | Chromodomain helicase DNA binding protein 5 isoform1 | 222685.25 | 5.79 | 0.02 | 7.12 | ATP binding |
| IPI00222188 | Collagen alpha-2(I) chain | 129556.97 | 9.27 | 0.17 | 2.05 | extracellular matrix structural constituent |
| IPI00620256 | Isoform A of Lamin-A/C | 74237.82 | 6.54 | 0.18 | 2.05 | ventricular cardiac muscle cell development |
| IPI00551341 | Eukaryotic translation initiation factor 4E nuclear import factor 1 | 25879.55 | 9.63 | 0.21 | 1.83 | translation initiation factor activity |
| IPI00621027 | Collagen alpha-2(VI) chain | 110334.44 | 6.01 | 0.27 | 1.55 | response to glucose stimulus |
| IPI00938530 | Myosin-11 isoform 1 | 223357.78 | 5.42 | 0.23 | 1.96 | smooth muscle contraction |
| IPI00316479 | Isoform 2 of Tyrosine-protein phosphatase non-receptor type 11 | 68034.75 | 6.87 | 0.16 | 2.95 | triglyceride metabolic process |
| IPI00653381 | PDZ and LIM domain protein 5 isoform ENH1 | 63299.28 | 8.61 | 0.28 | 1.69 | zinc ion binding |
| IPI00874362 | Putative uncharacterized protein, Lama2 | 342781.06 | 5.78 | 0.32 | 1.51 | regulation of cell adhesion and migration |
| IPI00880877 | Putative uncharacterized protein, Bola3 | 10828.61 | 8.51 | 0.24 | 1.97 | extracellular region |
| IPI00380895 | Myosin-3 | 223791.03 | 5.62 | 0.19 | 2.60 | structural constituent of muscle |
| IPI00130653 | Myosin-7 | 222879.18 | 5.59 | 0.23 | 2.19 | structural constituent of muscle |
| IPI00387388 | BAG family molecular chaperone regulator 3 | 61860.46 | 6.78 | 0.30 | 1.71 | anti-apoptosis |
| IPI00323035 | Fibulin-5 | 50193.55 | 4.55 | 0.29 | 1.77 | calcium ion binding |
| IPI00130102 | Desmin | 53497.99 | 5.21 | 0.30 | 1.70 | muscle organ development |
| IPI00387557 | Alpha-actinin-2 | 103833.9 | 5.31 | 0.30 | 1.70 | calcium ion binding |
| IPI00129404 | Myosin-6 | 223565.65 | 5.57 | 0.23 | 2.21 | structural constituent of muscle |
| IPI00312468 | Eukaryotic peptide chain release factor subunit 1 | 49030.89 | 5.51 | 0.13 | 3.84 | translation release factor activity, codon specific |
| IPI00775849 | Alpha-actinin | 59897.83 | 5.69 | 0.35 | 1.51 | cortical cytoskeleton |
| IPI00856603 | Putative uncharacterized protein, Klhl18 | 56405.27 | 5.48 | 0.31 | 1.78 | unclear |
| IPI00415685 | Isoform 3 of PDZ and LIM domain protein 5 | 26375.73 | 9.69 | 0.30 | 1.81 | zinc ion binding |
| IPI00116668 | Integrin-linked protein kinase | 51373.15 | 8.3 | 0.18 | 3.15 | ATP binding |
| IPI00114840 | Endonuclease G, mitochondrial | 32190.74 | 9.56 | 0.39 | 1.78 | cleaves DNA at double-stranded (DG)n |
| IPI00885372 | Mesdc2 Protein | 8380.56 | 4.78 | 0.36 | 1.54 | protein folding |
| IPI00111831 | Nascent polypeptide- associated complex subunit alpha, muscle- specific form | 220601.8 | 9.4 | 0.37 | 1.51 | DNA binding |
| IPI00828222 | Mutant fibrillin-1 | 418308.15 | 4.68 | 0.34 | 1.65 | calcium ion binding |
| IPI00756257 | Isoform 1 of Titin | 3906489.35 | 5.91 | 0.34 | 1.69 | adult heart development,  cardiac myofibril assembly |
| IPI00608008 | Isoform 2 of Nephrocystin-3 | 51835.99 | 5.92 | 0.26 | 2.20 | lipid metabolic process |
| IPI00604969 | Titin isoform N2-A | 3716017.14 | 6.03 | 0.35 | 1.65 | muscle contraction |
| IPI00273514 | Putative uncharacterized protein, Gm7806 | 18553.71 | 10.47 | 0.38 | 1.58 | structural constituent of ribosome |
| IPI00377642 | Pituitary tumor-transforming gene 1 protein-interacting protein | 19965.41 | 9 | 0.41 | 1.55 | protein import into nucleus |
| IPI00470167 | Reticulon-4 isoform B1 | 38404.08 | 4.7 | 0.33 | 1.92 | cardiac epithelial to mesenchymal transition |
| IPI00830606 | protein Pkp2 | 52491.34 | 9.58 | 0.23 | 2.86 | heart development |
| IPI00323030 | Isoform 1 of LIM domain-binding protein 3 | 76432.24 | 7.96 | 0.43 | 1.53 | zinc ion binding |
| IPI00555015 | Myosin regulatory light chain 2, ventricular/cardiac muscle isoform | 18864.35 | 4.86 | 0.38 | 1.73 | cardiac myofibril assembly, heart contraction |
| IPI00114375 | Dihydropyrimidinase-related protein 2 | 62277.51 | 5.95 | 0.40 | 1.67 | hydrolase activity |
| IPI00115823 | Myomesin 2 | 164737.89 | 5.54 | 0.37 | 1.80 | structural constituent of cytoskeleton |
| IPI00762303 | Putative uncharacterized protein, Ttc19 | 39716.59 | 5.99 | 0.29 | 2.33 | mitochondrial respiratory chain complex III assembly |
| IPI00785217 | Low-density lipoprotein receptor | 94947.38 | 4.82 | 0.22 | 3.05 | receptor activity |
| IPI00555113 | 60S ribosomal protein L18 | 21644.53 | 11.79 | 0.32 | 2.13 | structural constituent of ribosome |
| IPI00828837 | Isoform 4 of Protein CLEC16A | 100785.58 | 6.12 | 0.33 | 2.12 | unclear |
| IPI00751974 | Alpha-1-syntrophin | 53282.79 | 6.35 | 0.18 | 3.84 | phospholipid binding |
| IPI00128904 | Poly(rC)-binding protein 1 | 37497.77 | 6.66 | 0.31 | 2.27 | RNA binding |
| IPI00469840 | Pterin-4-alpha-carbinolamine dehydratase 2 | 14829.91 | 9.2 | 0.44 | 1.63 | 4-alpha-hydroxytetrahydrobiopterin dehydratase activity |
| IPI00125880 | Protein kinase C and casein kinase substrate in neurons protein 2 | 55832.88 | 5.1 | 0.46 | 1.56 | negative regulation of endocytosis |
| IPI00278864 | Peptide deformylase-like protein | 25779.31 | 9.35 | 0.35 | 2.05 | peptide deformylase activity |
| IPI00943405 | Isoform 2 of Microsomal triglyceride transfer protein large subunit | 100750.94 | 7.51 | 0.40 | 1.83 | lipid binding |
| IPI00749677 | Putative uncharacterized protein, Dnm2 | 97987.43 | 7.02 | 0.07 | 10.43 | GTP binding |
| IPI00626655 | Isoform 1 of Myomesin-1 | 185464.52 | 5.83 | 0.48 | 1.54 | muscle contraction |
| IPI00162942 | 39S ribosomal protein L37, mitochondrial | 48340.93 | 9 | 0.32 | 2.30 | structural constituent of ribosome |
| IPI00755365 | Glrx2 protein (Fragment) | 17620.15 | 9.43 | 0.38 | 1.96 | electron carrier activity |
| IPI00889927 | Ubiquitin-associated protein 2-like isoform 6 | 103945.98 | 6.49 | 0.38 | 1.96 | unclear |
| IPI00339916 | Bifunctional aminoacyl-tRNA synthetase | 170053.07 | 7.75 | 0.19 | 4.00 | ATP binding, RNA binding |
| IPI00127280 | Neutrophilic granule protein | 19331.74 | 5.21 | 0.24 | 3.24 | cysteine-type endopeptidase inhibitor activity |
| IPI00928138 | Septin 8 | 50895.75 | 5.74 | 0.47 | 1.66 | GTP binding |
| IPI00117846 | Death-associated protein kinase 3 | 51421.9 | 8.91 | 0.33 | 2.45 | ATP binding |
| IPI00134941 | Isoform 2B of GTPase KRas | 21482.49 | 8.25 | 0.50 | 1.59 | GTP binding |
| IPI00331286 | Beta-2-microglobulin precursor | 13778.95 | 8.55 | 0.39 | 2.04 | protein binding |
| IPI00113895 | Alpha-centractin | 42613.89 | 6.19 | 0.48 | 1.74 | ATP binding |
| IPI00895518 | Parkinson disease (Autosomal recessive, early onset) 7 | 18361.2 | 6.09 | 0.47 | 1.80 | RNA binding |
| IPI00655029 | Sorbin and SH3 domain-containing protein 1 isoform 2 | 103977.89 | 5.65 | 0.46 | 1.83 | protein kinase binding |
| IPI00828253 | Dystrophin | 425831.77 | 5.66 | 0.46 | 1.88 | muscle cell homeostasis |
| IPI00930882 | 4F2 cell-surface antigen heavy chain isoform a | 62239.78 | 5.32 | 0.52 | 1.69 | carbohydrate metabolic process |
| IPI00136293 | Transmembrane protein C9orf46 homolog | 17261.18 | 9.48 | 0.53 | 1.68 | positive regulation of plasminogen activation |
| IPI00124411 | Protein-tyrosine phosphatase-like member B | 28402.43 | 9.59 | 0.23 | 3.95 | fatty acid biosynthetic process |
| IPI00313884 | Podocalyxin-like protein 1 | 53389.21 | 4.86 | 0.51 | 1.77 | cell adhesion |
| IPI00830858 | Popeye domain- containing protein 2 isoform 1 | 41847.21 | 4.79 | 0.60 | 1.51 | unclear |
| IPI00918897 | Putative uncharacterized protein, Nebl | 51691.35 | 6.39 | 0.43 | 2.12 | unclear |
| IPI00331443 | Farsb Putative uncharacterized protein | 65815.42 | 7.16 | 0.58 | 1.57 | ATP binding |
| IPI00408218 | Zyx protein | 57027.19 | 6.2 | 0.18 | 5.10 | protein binding |
| IPI00128345 | NADH dehydrogenase [ubiquinone] iron-sulfur protein 6, mitochondrial | 13019.78 | 8.86 | 0.59 | 1.55 | electron transport |
| IPI00911157 | Troponin T, cardiac muscle isoform c | 35498.27 | 4.98 | 0.58 | 1.58 | regulation of heart contraction |
| IPI00225747 | Rik Transmembrane protein C3orf1 homolog | 31791.2 | 9.32 | 0.56 | 1.67 | unclear |
| IPI00881968 | Putative uncharacterized protein, Vps29 | 13723 | 7.8 | 0.42 | 2.21 | hydrolase activity, acting on ester bonds |
| IPI00137907 | Rik Putative uncharacterized protein | 11901.92 | 9.91 | 0.13 | 7.13 | unclear |
| IPI00320188 | Nicotinamide phosphoribosyltransferase | 55446.82 | 6.69 | 0.53 | 1.78 | nicotinamide phosphoribosyltransferase activity |
| IPI00129134 | Ly6c2 lymphocyte antigen 6C2 precursor | 14192.52 | 5.75 | 0.55 | 1.74 | anchored to membrane |
| IPI00929796 | 5'-AMP-activated protein kinase catalytic subunit alpha-2 | 62022.37 | 7.94 | 0.43 | 1.81 | an energy sensor protein kinase that plays a key role in regulating cellular energy metabolism |
| IPI00119680 | AP-1 complex subunit mu-1 | 48542.9 | 6.82 | 0.61 | 1.55 | intracellular protein transport |
| IPI00751087 | Dolichol-phosphate (Beta-D) mannosyltransferase 1 | 23432.19 | 9.32 | 0.41 | 2.35 | dolichyl-phosphate beta-D-mannosyltransferase activity |
| IPI00759948 | Isoform 2 of Gelsolin | 80762.65 | 5.52 | 0.41 | 2.33 | actin binding |
| IPI00315550 | Glutaredoxin-3 | 37778.42 | 5.42 | 0.35 | 1.61 | regulation of cardiac muscle hypertrophy and heart contraction |
| IPI00133167 | Gm9803 Mitochondrial import inner membrane translocase subunit Tim16 | 13784.66 | 9.64 | 0.59 | 1.64 | protein transport |
| IPI00420996 | Isoform 4 of Voltage-dependent L-type calcium channel subunit beta-2 | 64714.53 | 9.17 | 0.59 | 1.69 | high voltage-gated calcium channel activity |
| IPI00109221 | Phosphatidylinositide phosphatase SAC1 | 66943.8 | 6.85 | 0.56 | 1.78 | phosphatase activity |
